# Description of a Human Male Monster, with Remarks

**Published:** 1797

**Authors:** Alexander Monro

**Affiliations:** Fellow of the Royal College of Physicians, Professor of Medicine, Anatomy, and Surgery in the University of Edinburgh, Fellow of the Royal Academy of Surgery in Paris, &c.


XVI. *Defcription of a Human Male Monfter, with Remarks. By* Alexander Monro, *M.D. F.R.S. Edin. Fellow of the Royal College of Phyficians, Profeffor of Medicine, Anatomy, and Surgery in the Univerfity of Edinburgh, Fellow of the Royal Academy of Surgery in Paris,* &c. Vide *Tranfactions of the Royal Society of Edinburgh.* Vol. III. 4to. Edinburgh, 1794.

THE monfter here defcribed, of which the mother was delivered after the birth of a complete child at the full time, had its proper membranes and a placenta, with a fhort umbilical cord.

The following parts were wanting in it, viz. the bones of the head; the brain, with the organs of fight, hearing, fmell, and tafte; the neck; about one half of the ribs; the larynx, trachea, and lungs; the heart; the pharynx, œfophagus, and ftomach, with all the fmall inteftines, except the end of the ilium; the anus; the liver, fpleen, pancreas, and omenta; the renal glands; termination of the ureters; both both arms; both patellæ; and ſeveral of the bones of the feet and toes.

A round opening, which led to a thimble-like cavity, ſhut at its bottom, had ſome diſtant reſemblance to the mouth.

The ſoft parts of the trunk were ſupported by ſixteen vertebræ, ſix ribs, an os ſacrum, and two oſſa innominata. The legs had each an os femoris, tibia, and fibula, with an imperfect number of the bones of the feet.

The umbilical cord was connected at nearly the uſual height above the oſſa pubis.

The penis, covered with a large preputium, had the uſual ſituation and ſtructure.

The lower part of the trunk contained an inteſtinal tube, ſhut at its beginning, and compoſed of an upper part, four inches long, reſembling the end of the ilium; for it terminated in the ſide of an inteſtine, reſembling the caput coli, with its appendix vermiformis. From this place, to its lower end, the great inteſtine meaſured thirteen inches; and the end of the rectum terminated in the back part of the urinary bladder. The rectum contained a viſcid ſemipellucid mucus; but nothing like the meconium.

In the meſentery and meſocolon, there were about about a dozen conglobated lymphatic glands, of the ufual fhape, colour, and confiftence; from which it appeared that the inteftines were provided with lacteal veffels, and it is therefore, obferves our author, not to be doubted that the other parts of the body were furnifhed with lymphatics, or that there was an abforbent as well as circulating fyftem in this monfter.

At the upper part of the trunk, covered by the ribs, there were two kidnies of a large fize, with a pelvis and ureter to each. Both were fhut at their lower ends, and had no communication with a fmall fac, which, in fituation and ftructure, refembled the urinary bladder, and had an urachus coming from it.

There was only one teftis, fituated, in the ufual manner, on the left fide.

The urethra was wanting from within an inch of the urinary bladder to within an inch of the extremity of the penis.

The fpinal marrow was of a conical fhape, with the top or fmall part of the cone at its upper end, and at its lower end it formed a cauda equina. From its two ends and fides it fent off eighteen pair of nerves, which, at their origin and in their progrefs, we are told, were nearly as large as in a perfect fœtus.

The

The umbilical chord, Dr. Monro obſerves, was nearly proportioned to the bulk of the monſter; and, at the umbilicus, conſiſted of one vein and two arteries, within which he found red blood. The vein, he adds, was more capacious than both arteries together; and as ſoon as it entered the abdomen, was divided into various branches, which were diſperſed upon all parts of the body.

Veſſels, our author remarks, every where accompanied the branches of the umbilical vein, correſponding with them in ſize as well as ſituation; and joining together, formed trunks, from which, at the ſides of the pelvis, two veſſels were continued, one of them on each ſide of the veſica urinaria and urachus, to the umbilicus, which they perforated, and then went, along the umbilical cord, towards the placenta, reſembling the umbilical arteries.

Dr. Monro regrets, that before he received this monſter, the injection of its placenta had been intruſted to ſome perſon who had managed it ſo negligently, that nothing could be determined as to the diſtribution or communication of the veſſels of the placenta with each other, or with thoſe of the placenta of the complete child, or with thoſe of the mother.

We

We muſt refer our readers to the work itſelf for four tables illuſtrative of this deſcription, all the figures of which are of the natural ſize. One of theſe figures *, which repreſents the fore view of it entire, we have taken the liberty to copy on a reduced ſcale of one third of the ſize of the original engraving, for the ſake of conveying to our readers a more accurate idea of this ſingular production than can poſſibly be derived from a verbal deſcription alone.

We come now to the very judicious and intereſting remarks of the learned author on productions of this kind. Theſe remarks are arranged under diſtinct heads or ſections. The firſt conſiſts of preliminary obſervations. In the ſecond and third ſections he treats of the direction and the cauſes of the motion of the blood in this monſter; in the fourth he conſiders its nervous ſyſtem; in the fifth, the duration of its life; and in the ſeventh and laſt ſection he conſiders the time at which this monſter

* See Plate II. fig. 3, in which *a, b, c,* refer to a circular maſs, more than two inches thick, which ſupplied the place of head, trunk, and arms; *d,* to a thimble-like cavity, ſomewhat reſembling the mouth; *e,* to the umbilical chord; *f, g,* to the penis and preputium; and *h, i, k, l, m, n,* to the thighs, legs, and feet.

muſt muſt have acquired the ſtructure which has been deſcribed.

Monſters wanting the head, heart, and lungs, and in almoſt every other reſpect agreeing with that above deſcribed, have, he obſerves, been mentioned by different writers, particularly by Mery and Winſlow *; and Roederer †, he adds, has given a full deſcription of a monſter, in which one ſmall muſcular ſac only was found, inſtead of a complete heart, communicating with the continuation of one of two veins which were found in the umbilical cord; but our author is of opinion that the real courſe of the blood, or the cauſes of its motion, have been miſapprehended by all thoſe writers.

Mery, he obſerves, thinks the blood of the fœtus muſt have been moved by the motion of the heart of the mother, and conſiders the want of the heart in ſuch monſters, as a ſtrong confirmation of the opinion he entertained, that there is a circulation of the blood carried on between the mother and the fœtus ‡.

Winſlow,

* Mem. de l'Acad. des Sciences, 1720 and 1740.

† Comment. Societ. R. Scient. Gotting. Tom. IV. 1754.

‡ Mery, Mem. de l'Acad. des Scien. 1720. 1re Reflexion. " Sa vie n'a pu avoir pour principes que la reſpi-

" ration

Winflow, remarks our author, from not having found any red blood in the veffels of the fœtus, nor traced within it the branches of the umbilical vein, but thofe only, as he imagined, of the veffel he called aorta, and which he thought performed the office of an artery, was led to the fuppofition, that, inftead of a circulation, there was only a fort of progreffion of the colourlefs blood, or lymphatic humour, to the capillary extremities of the arterial ramifications, and that it tranfuded, by little and little, and very flowly, into the cellular texture of all the parts, and perhaps, at laft, paffed through the pores of the fkin, in form of moifture*.

Dr.

" ration et le mouvement circulaire du fang de fa mere." And in the Hiftorical part of the fame volume, " Le defaut " du cœur prouve que le fang qui a circulé dans ce fœtus " ne recevoit fon impulfion que du cœur de la mere.... " M. Mery a toûjours foutenu la circulation reciproque " entre la mere et le fœtus, et telle que le fœtus eft toujours " comme un membre de la mere."

* Winflow, Mem. de l'Acad. des Scien. 1740.

P. 588. " La veine ombilicale, s'étant écartée du cor- " don dès fon entrée dans le ventre, y formoit un tronc fort " court, qui montoit tout droit, et s'implantoit à la bafe du " bouton cutané, *s'adoffant* là avec le tronc d'un autre vaif-

" feau

Dr. Roederer* not only applies the term of vena cava to the large vein with which the umbilical vein is joined to the heart, but defcribes the cava as afcending from the abdomen to the

" feau de pareille groffeur, qui fortoit de la même bafe, et
" qui etant d'abord courbé vers en bas, defcendoit derriere
" le paquet des inteftins, à peu près comme le tronc de la
" portion inferieure de l'aorte, et fe diftribuoit enfuite en
" plufieurs branches, de la maniere que je dirai ci après."

P. 590. " On ne voyoit pas une goutte ni aucune ap-
" parence de fang rouge dans toute l'étenduë du corps de
" cet enfant, ni aucun veftige de vaiffeaux veineux."

P. 600. " Hors la petite portion de la veine ombilicale
" après fon entrée par le nombril, je n'ai trouvé dans tout
" le corps de cet enfant aucun vaiffeau veineux, ni le
" moindre veftige, foit de tronc, foit de ramifications de
" veines."

P. 604. " Mais à l'egard de la circulation intrinfeque
" dans les parties mêmes de ce demi-corps, l'abfence, ou la
" privation totale, de vaiffeaux veineux m'a fait conjecturer,
" qu'au lieu de circulation proprement dite, il n'y a eu
" qu'une efpece de progreffion ou trufion jufqu'aux extre-
" mités capillaires de toutes les ramifications arterielles, et
" que là ce fang lymphatique tranfudoit peu à peu, et très
" lentement dans le tiffu cellulaire de toutes les parties....
" Et, peut-être, paffoit par les pores externes de la peau
" en maniere de moiteur. Je n'avance tout ceci que comme
" des pures conjectures."

* Com. Soc. R. Sc. Gotting. tom. iv. com. 4.

 thorax.

thorax *. In like manner, he not only applies the name aorta to the veſſel which accompanies the continuation of the umbilical veins; but ſpeaks of the aorta as aſcending from the thorax to the head †, and ſending off the ſubclavian and carotid arteries; and remarks, that canals proper to the latter were wanting ‡. And he obſerves, that the aorta, after deſcending, as uſual, between the crura of the diaphragm,

* P. 142. " Duplicem autem umbilicalis funis venam " largitur. Altera minor.... cum vena cava, ex abdomine aſ- " cendente confluit."

† P. 144. " Arteria magna, quam aortam vocant....ex " abdomine in thoracem aſcendit. In thorace eandem pene " directionem.... ſervans, nulloque cum corde canali con- " fluens, ſola et a corde diſtincta, iter ſuum abſolvit. Nullus " proinde ex aorta arcus formari poteſt, ſed laterales rami " ex recto aortæ trunco emittuntur. Sunt iſti rami qui " deſcripti ſequuntur.

" In regione coſtæ primæ leviſſime deſcendentes arteriæ " ſubclaviæ naſcuntur, ex quibus viciſſim triplex alia ra- " morum ſpecies oritur; quarum prima ad cervicis muſcol- " los aſcendit. Porro truncus aortæ per ſemi pollicem poſt- " quam progreſſus eſt in duos ramos dividitur, duas nempe " arterias carotides, quæ ad altitudinem laryngis ſine in- " ſigniori ramo aſcendunt.... Aſcendit autem carotis dex- " tra, &c.—Ad latus tandem laryngis carotis communis in " ſex omnino ramos dividitur."

‡ P. 166. " Canalis pro arteria carotide deeſt. Carotis " per amplum foramen lacerum ad cerebrum tendit."

gave gave off the mesenteric, renal, lumbar, and iliac arteries; and that the left iliac artery sent off an umbilical artery: and concludes his description in the following words: "Ita quidem, si arteriæ umbilicalis dextræ, arteriæque cæliacæ defectus.... excipiatur, vix ab usitata fabrica aberrans arteria aorta in abdomine distribuitur *."

After an elaborate description of the several parts of the monster, Dr. Roederer proposes the cause of the motion of its humours, in the following words:

"Motus qui .... humores agitat, causa indagatur .... Ast aliquis, lentus licet, parasitici fetus humores motus agitavit.... A corde, sueto motore, repeti iste motus nequit, neque multum auxilii propulsus in uterum maternum sanguis ferre potest. Præter vero istum levem debilemque.... Ipsa vasorum actio, sive contrahendo agat, sive attrahendo, vi illa capillaribus tubis familiari, præcipuum humoribus motum impertiri debet.... Accedunt forsan et aliæ in fetu nostro causæ incognitæ, ipsa fortasse a calore excitata fluidorum agitatio aliaque †."

* Comm. Soc. R. Scient. Gotting. Tom. IV. p. 155.
† Ibid. p. 212.

But as to the direction in which he ſuppoſed the humour to be moved, Dr. Roederer, obſerves our author, ſays nothing, and therefore leaves the reader to judge of his opinion, from the foregoing deſcription of the blood veſſels.

Dr. Monro thinks, that to the opinions of all theſe authors, when fully conſidered, we ſhall find inſuperable objections Thus, without ſaying in objection to that of Mery, that it is ſo far from being certain, that there is a circulation of red blood between the mother and fœtus, that the contrary opinion is the moſt probable, we cannot, he obſerves, conceive, although the anaſtomoſes of the uterine with the placentary veſſels were proved, that the mere impulſe of the blood in the minute arteries ſhould have carried the blood, not only into the trunks, but through all the capillary branches of the veſſels of the fœtus, and again back from theſe to the placenta, and from its umbilical arteries into the umbilical veins and veins of the uterus.

Dr. Monro contends, that the opinion of Winſlow is far more unſatisfactory than that of Mery. He obſerves, that in the firſt place, it cannot be applied to the monſter deſcribed by Mery, or to that which is the ſubject of the preſent paper, where there were two ſets of veſſels; vessels; and, in the next place, that Winslow was so far from tracing distinctly the joining of the umbilical vein with the vessel he calls aorta, that he describes it as merely *s'adossant* with the trunk of the aorta*; and farther, that although he repeatedly affirms there were no venous vessels in any part of the body of the monster, yet his description of the vessels of the kidney will not be found to correspond with his general assertion; for he describes a vessel which, indeed, he calls arterious, but which began on the fore part of the belly above the navel, at the place where the small portion of the umbilical vein terminated in the cavity of the cutaneous button, from which various branches were sent into the kidney at its convex part, and from its concave part different arteries, he says, came out in an extraordinary manner †.

* See Mem. de l'Acad. des Sciences, 1740, p. 588; or the note in page 176 of the present volume.

* P. 602. "Ce tronc artériel qui étoit comme la portion inferieure de l'aorte descendante, au lieu de tenir la route naturelle en arriére le long des vertebres, en étoit ici très eloigné. Il commencoit sur le devant du ventre au dessus du nombril, à l'endroit où se terminoit la petite portion de la veine ombilicale.... Il jettoit des branches dans la masse du rein par ....... sa convexité. Il sortoit de la concavité plusieurs artères."

Upon the whole, as the umbilical cord is not ſaid to have been uncommon in ſize or ſtructure; as there were two ſorts of veſſels connected with the kidney; as it is ſo improbable, as to be incredible, that the fœtus received arteries without correſponding veins, or that there was merely a protruſion of the humours, and exudation of them, without circulation, Dr. Monro has no doubt that Winſlow, eſpecially as he did not inject the veſſels of the umbilical cord, has miſtaken the continuation of the umbilical veins, and the branches of the veſſels he calls aorta, for branches of the ſame veſſel; and as the monſter he examined agreed very nearly, in all other reſpects, with that which is the ſubject of the paper before us, our author apprehends it muſt have agreed likewiſe in having two kinds of blood-veſſels, arteries, and veins.

Dr. Roederer rejects the opinion of Mery, that the blood of the fœtus is circulated by the heart of the mother, and ſuppoſes, that capillary attraction, heat, and ſome activity of the veſſels, may contribute to its motion. But as he applies the term aorta, not to the continuation of the umbilical vein, but to the other principal veſſel of the monſter, and deſcribes it as ſending branches downwards from the abdomen to the inferior extremities, and upwards from the thorax to the head, and applies the name of carotid arteries to two of theſe branches, with the additional remark, that the canales carotici were wanting, it will, our author thinks, appear evident from theſe circumſtances, and from what he obſerves in his ſecond ſection, to which we now come, and in which he conſiders the direction of the blood in this monſter, that Dr. Roederer has miſtaken the direction in which the blood was moved and circulated.

As there were two kinds of veſſels, arterious and venous, in the umbilical cord, and likewiſe within the body of this monſter, it cannot, our author thinks, be doubted that theſe communicated with each other, and that the blood was conveyed by them in a circle.

To deſcribe the circle more exactly, we cannot, he adds, doubt that the blood was conveyed from the placenta by the umbilical vein into the body of the monſter. We next found, he obſerves, that the umbilical vein within the monſter was divided into various branches, which could be traced to all its parts, or that theſe branches performed the office of arteries, or resembled the vena porta hepatica; and that contiguous to these branches were found, every where, other vessels forming a trunk or large vessel, which, by its situation, resembled our aorta: but we must suppose, continues our author, that these branches served the purpose of receiving the blood from the extremities of the branches of the umbilical vein, or were in reality venous vessels. From the vessel resembling the aorta in situation, but very different in office, two vessels were sent off, which ran at the sides of the bladder to the umbilicus, and formed the arteries of the umbilical cord and of the placenta; and, in the placenta, must have terminated in the minute beginnings of the umbilical vein, to complete the circle in which the fœtal blood was moved.

Thus, he adds, we find the umbilical vein in the placenta and umbilical cord performing the office of a vein, while its continuation within the body of the monster performed the office of an artery; and, on the other hand, we see the vessel he has called aorta, performing the office of a vein within the monster, and that of an artery in the umbilical cord and placenta.

Dr. Monro next considers the causes of the motion of the blood in this monster. In that, he he obſerves, which was examined by Winſlow, and which appears to have agreed very nearly with the one here deſcribed, no red blood was found in any of the veſſels; and therefore we muſt conclude, that none of the red arteries of the mother anaſtomoſed with the umbilical veins; and even where this is the ordinary ſtructure, it is ſo far, adds our author, from being certain that the veſſels of the uterus, which contain red blood, anaſtomoſe with thoſe of the umbilical cord, that the contrary is the moſt probable opinion. He therefore conſiders it as very improbable that the blood in the umbilical vein was puſhed on by the heart of the mother; and even were we to admit that the arteries of the mother anaſtomoſed with the umbilical veins, yet as their communications muſt have been very minute, and the momentum of the blood in them very much broken, we cannot, he thinks, conceive that it could have been ſufficient to puſh the blood through the terminations of all the branches of the umbilical veins, in the ſeveral organs of its body, into the veſſel we call aorta, and again from the aorta back to the placenta by the umbilical arteries, and through the minute branches of theſe thefe to the veins of the mother, and beginnings of the umbilical veins.

Hence our author is led to conclude that the circulation of the blood in the placenta and body of the monfter was carried on by a well-regulated mufcular action of the blood veffels. In the *echinus efculentus* he has found in the mefentery, which is a principal part of it, two fuch large veffels without a heart, and which, as he obferves, we can fcarcely doubt, refembled our aorta and vena cava, and circulated its fluid; and in fifhes *, he adds, the blood which paffes through the liver defcribes three circles, and in all other parts of the fifh the blood defcribes two circles before it returns to the heart; which motion of it we muft fuppofe to be chiefly owing to the mufcular action of the veffels, as the force of the heart appears to be as much fpent in the gills of the fifh as in the lungs of a man.

From confidering the manner and caufe of the motion of the blood in this monfter, and comparing with it the motion of the blood in fifhes, and in the fea egg, our author is, by

* See Monro on Fifhes, p. 67. tab. xliii.

analogy, led to the following general conclusions:

1. That the arteries contribute much to the circulation of the blood in our bodies.

2. That it is probable that, in man, the veins likewife affift in circulation; and, in particular, that there can be no doubt that the vena portarum, by its action, contributes much to the motion of the blood through our liver.

3. That for the like reafons, we may conclude, that arterious veffels, independently of the impulfe of the heart, may act in fuch a manner, as to perform the fecretion of liquors, to nourifh the folids, and to add to their bulk; and particularly, that the branches of the vena portarum change certain parts of the blood into bile.

We come now to the author's remarks on the nervous fyftem of this monfter.

As the fpinal marrow, and pairs of nerves fent off from it, had nearly the ufual fize and ftructure, although the brain, cerebellum, and medulla oblongata were entirely wanting, he finds reafon for calling in queftion the common doctrine of authors, which teaches, that the fpinal marrow and nerves derive their origin from the brain and cerebellum, and are dependent upon it it as much as the ducts of the glands are upon the glands which fend liquors into them.

As the feveral parts of the monfter were furnifhed with nerves, and we have feen that its arteries and veins, by a well-regulated, varied, and complicated action, circulated the blood, we muft, he contends, fuppofe that their mufcular fibres were actuated by thofe nerves. We therefore, he obferves, find in this monfter not only the exiftence and common appearance of the fpinal marrow and nerves connected with it, although the brain and cerebellum were wanting; but we learn from it that thefe, independently of the brain and cerebellum, may actuate the mufcular fibres in the veffels of an animal; or, in other words, that nervous energy, or fluid, as it is commonly called, is not derived from the brain and cerebellum folely; but that the nerves alfo are capable of furnifhing it: from all which, he thinks, it follows, that there is no more reafon for believing that the nerves are derived from the brain, than that the latter is derived from the nerves; and, of courfe, that all the parts and branches of the nervous fyftem appear to poffefs the general power or office of furnifhing nervous energy.

With refpect to the duration of the life of this this monſter, our author remarks, that as in man and ſimilar animals, the direct or indirect influence of reſpiration ſeems neceſſary for the continuance of life, and as the lungs were wanting in this monſter, it muſt be ſuppoſed, that it could have outlived the ſeparation from the mother for a very ſhort time only; and when we farther conſider, that, by the ligature of the umbilical cord, a ſtop would be mechanically put to the circulation of its blood, it is evident, he adds, that its life muſt have terminated with its delivery.

Dr. Monro concludes this inſtructive paper with obſerving, that as this monſter was provided with a diſtinct placenta and membranes, and its body ſurrounded with and protected by the liquor amnii; as no veſtige appeared of the brain, cerebellum, organs of the ſenſes, or other parts of the head; as nervous threads, proper to this monſter, aſcended from the upper end of the ſpinal marrow towards the upper parts of its body; as its ſyſtem of circulating veſſels was complete without a heart, and the manner of their branching different in many reſpects from the common ſtructure: it muſt ſurely appear, to an unprejudiced perſon, abſurd to ſuppoſe, with many eminent authors, that that ſuch monſters, when firſt produced, had the ordinary ſtructure, and that this was afterwards altered by preſſure and other accidents.

A ſimilar obſervation, he thinks, may be extended not only to many other monſters in his poſſeſſion, but (as he believes he might ſay) to almoſt all other monſters which have been deſcribed; particularly to two, of which he has given a deſcription, illuſtrated with figures, in his valuable work on the Nervous Syſtem (Table VIII.** and Tab. XII.). In one of them, a human monſter, one heart ſupplied two heads and two trunks; in the other, a kitten, one heart, conſiſting of two auricles and two ventricles, ſent off from its left ventricle one aorta, which ſupplied one head and two bodies.

